# The unique electrical properties in an extracellular fluid of the mammalian cochlea; their functional roles, homeostatic processes, and pathological significance

**DOI:** 10.1007/s00424-016-1871-0

**Published:** 2016-08-27

**Authors:** Fumiaki Nin, Takamasa Yoshida, Seishiro Sawamura, Genki Ogata, Takeru Ota, Taiga Higuchi, Shingo Murakami, Katsumi Doi, Yoshihisa Kurachi, Hiroshi HIBINO

**Affiliations:** 1 Department of Molecular Physiology, Niigata University School of Medicine, 1-757 Asahimachi-dori, Chuo-ku, Niigata, Niigata 951-8510 Japan; 2 Center for Transdisciplinary Research, Niigata University, Niigata, 950-2181 Japan; 3 Department of Otorhinolaryngology, Graduate School of Medical Sciences, Kyushu University, Fukuoka, 812-8582 Japan; 4 Division of Molecular and Cellular Pharmacology, Department of Pharmacology, Osaka University, Osaka, 565-0871 Japan; 5 Center for Advanced Medical Engineering and Informatics, Graduate School of Medicine, Osaka University, Osaka, 565-0871 Japan; 6 Department of Physiology, School of Medicine, Toho University, Tokyo, 143-8540 Japan; 7 Department of Otolaryngology, Kindai University Faculty of Medicine, Osaka, 589-8511 Japan; 8 AMED-CREST, AMED, Niigata, Japan

**Keywords:** Cochlea, Hearing, Endocochlear potential, Ion concentrations, K+ transport, Electrophysiology, Computer simulation

## Abstract

The cochlea of the mammalian inner ear contains an endolymph that exhibits an endocochlear potential (EP) of +80 mV with a [K^+^] of 150 mM. This unusual extracellular solution is maintained by the cochlear lateral wall, a double-layered epithelial-like tissue. Acoustic stimuli allow endolymphatic K^+^ to enter sensory hair cells and excite them. The positive EP accelerates this K^+^ influx, thereby sensitizing hearing. K^+^ exits from hair cells and circulates back to the lateral wall, which unidirectionally transports K^+^ to the endolymph. In vivo electrophysiological assays demonstrated that the EP stems primarily from two K^+^ diffusion potentials yielded by [K^+^] gradients between intracellular and extracellular compartments in the lateral wall. Such gradients seem to be controlled by ion channels and transporters expressed in particular membrane domains of the two layers. Analyses of human deafness genes and genetically modified mice suggested the contribution of these channels and transporters to EP and hearing. A computational model, which reconstitutes unidirectional K^+^ transport by incorporating channels and transporters in the lateral wall and connects this transport to hair cell transcellular K^+^ fluxes, simulates the circulation current flowing between the endolymph and the perilymph. In this model, modulation of the circulation current profile accounts for the processes leading to EP loss under pathological conditions. This article not only summarizes the unique physiological and molecular mechanisms underlying homeostasis of the EP and their pathological relevance but also describes the interplay between EP and circulation current.

## Introduction

The auditory system continuously receives sounds of different intensities and frequencies from the ambient and extracts information necessary for the organism. The cochlea of the inner ear, the peripheral organ for hearing, harbors sensory hair cells that transduce mechanical stimuli to electrical signals. In humans, the audible frequencies range from 20 to 20,000 Hz, whereas the maximum hearing threshold is a trillionfold larger in acoustic power than the minimum threshold [44]. These striking profiles depend not only on the properties of hair cells but also on the ones of a unique extracellular solution that immerses them [14, 22, 36, 42, 109]. This solution is called “endolymph.”

**Fig. 1:**
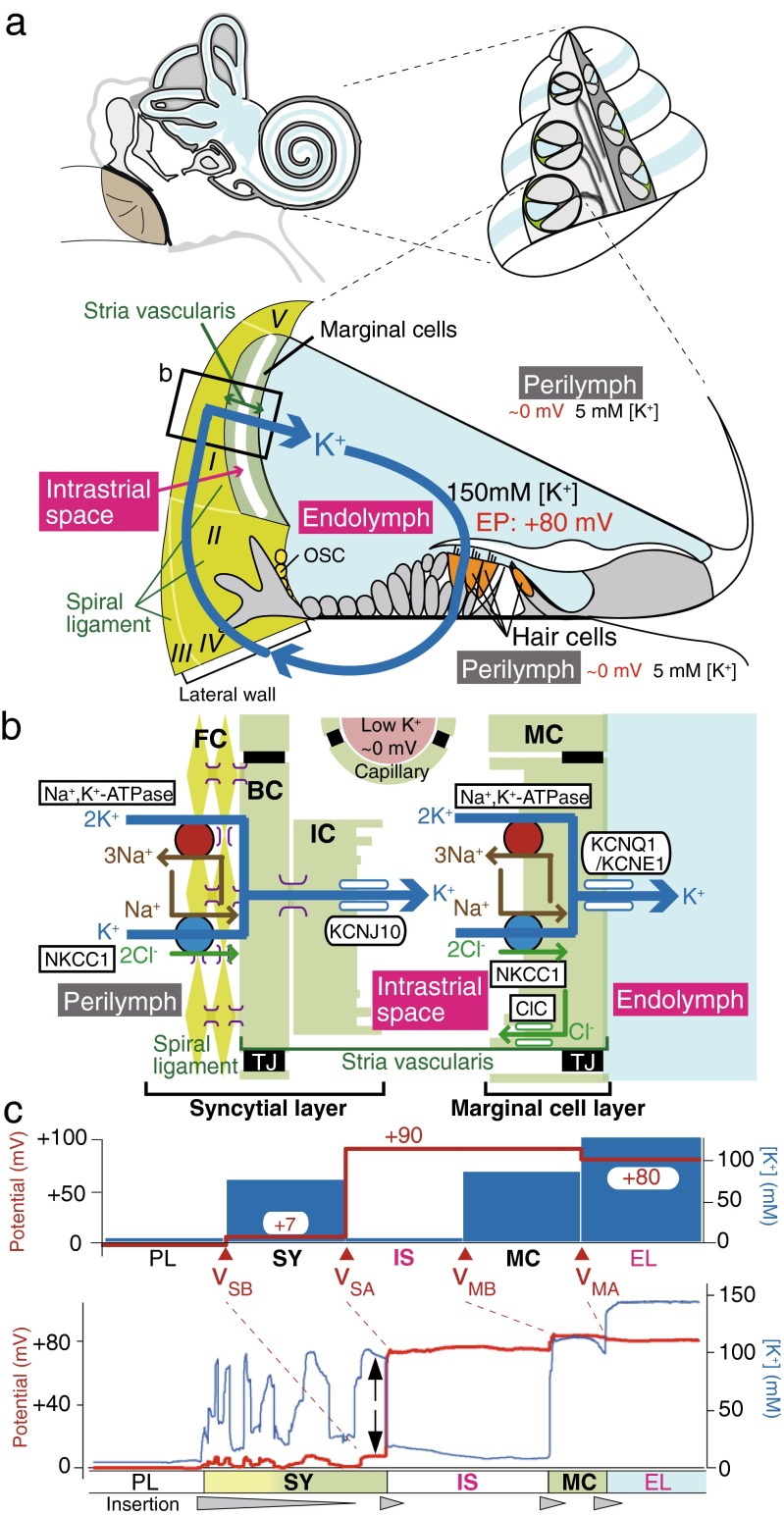
Electrochemical profile of the cochlea. **a** Structure of the cochlea. The composition of the human ear and a cross section of the cochlea are illustrated in the *upper left* and *right panels*, respectively. The *lower panel* depicts the tissue and cellular components of the cochlea. The electrochemical properties of the endolymph and perilymph and the possible circulation of K^+^ between the two are also shown. Note that the intrastrial space in the stria vascularis is emphasized with an expansion, although it is a narrow extracellular space of 15 nm in width. The locations of the five types of fibrocytes in the spiral ligament are indicated by *roman numerals*. The *boxed region* is enlarged in **b**. *EP* endocochlear potential, *OSC* outer sulcus cells. Reproduced and modified from [74]. **b** Cellular components of the lateral wall. Ion channels and transporters that are likely to play key roles in the maintenance of unidirectional K^+^ transport across the lateral wall and endocochlear potential (EP) are shown in the *upper panel*. The lateral wall comprises the marginal cell layer and the syncytial layer that results from interconnections between fibrocytes (FCs) in the spiral ligament and the basal and intermediate cells (BC and IC, respectively) in the stria vascularis. Note that the basolateral and apical surfaces of the syncytial layer are provided primarily by fibrocytes and intermediate cells, respectively. *MC* marginal cells, *NKCC1* Na^+^,K^+^,2Cl^−^-cotransporter type 1, *ClC* ClC-K/barttin type Cl^−^ channel, *TJ* tight junction. Reproduced and modified from [74]. **c** Potential and [K^+^] properties of the lateral wall under physiological conditions. The *top panel* displays the properties averaged from multiple measurements. *v*_SB_, *v*_SA_, *v*_MB_, and *v*_MA_ indicate the membrane potentials across the basolateral and apical surfaces of the syncytial layer and the basolateral and apical surfaces of the marginal cell layer, respectively. Shown in the *lower panel* is a representative trace of the measurement in the cochlea of a live guinea pig. In this experiment, a microelectrode sensitive to potential (*red*) and [K^+^] (*blue*) was inserted from the perilymph (PL) into the endolymph (EL) across the lateral wall. Potentials in each compartment were measured with respect to the PL, which is defined to be 0 mV in in vivo assays. Spike-like variations of [K^+^] represent the passage of numerous fibrocytes interconnected with each other by gap junctions. Within this region, the compartment located next to the intrastiral space (IS) likely represents the properties of the syncytial layer (SY) (i.e., *v*_SB_ and [K^+^] inside the syncytium; indicated by *arrows*) [1, 116]. The *wedges filled with gray* below the *lower panel* indicate the periods during which the electrode was inserted toward the EL. *MC* marginal cells. Reproduced and modified from [115]

The cochlea is filled with the following two different extracellular solutions: perilymph and endolymph. Textbooks for life science such as “Molecular Biology of the Cell” and “Principles of Neural Science” explain that, in general, extracellular solutions including blood plasma, interstitial fluid, and cerebrospinal fluid bear a [K^+^] of 5 mM and a [Na^+^] of 150 mM [3, 51]. This ionic composition is conserved in the perilymph. On the other hand, the cochlear endolymph contains 150 mM K^+^ and 5 mM Na^+^ (Fig. 1a) [91, 94]. Furthermore, this solution exhibits an endocochlear potential (EP) of +80 mV with respect to the perilymph [5, 107]. Hair cells expose stereocilia extruded from their apical surface to the endolymph and bath their basolateral membranes in the perilymph. Acoustic stimuli open mechanoelectrical transduction (MET) channels at the top of the stereocilia, facilitating the entry of K^+^ from the endolymph into hair cells. This K^+^ influx depolarizes cells. Influx of Ca^2+^, which is also contained at a concentration of approximately 20 μM in the endolymph, simultaneously occurs and tunes the hair cell functions involved in establishing the aforementioned dynamic ranges of audition [43]. The EP greatly enhances these ionic flows by increasing the driving force and thereby crucially contributes to the achievement of superior profiles of hearing [14, 22, 42, 46]. Loss of EP causes deafness [42, 59]. After electrically exciting hair cells, K^+^ is likely to exit from the basolateral membranes and reach the perilymph through K^+^ channels (Fig. 1a) [20, 109].

The EP was measured for the first time in 1952 by Georg von Békésy, who won the Nobel Prize for the discovery of the physical mechanism of stimulation within the cochlea [5, 107]. Tasaki et al. (1959) found that the stria vascularis in the lateral cochlear wall is the source of the EP (Fig. 1a) [103]. Given that the stria harbors abundant vasculature, the maintenance of the EP was thought to need high energy. Indeed, anoxia causes a marked decrease in the EP [59, 63, 64]. Moreover, in vivo experiments showed that K^+^ is actively supplied to the endolymph from the perilymph, rather than from the blood [60]. Substitution of a solution containing a low [K^+^] with the perilymph markedly reduced the EP [61, 89]. Accordingly, it was believed that the lateral wall, which consists primarily of the stria and the neighboring connective tissue, the spiral ligament, constitutes a route for the transport of K^+^ from the perilymph to the endolymph and that this ionic flow is crucial for the maintenance of the unique properties of the endolymph (Fig. 1a, b) [55, 56, 108, 109]. Several physiological, histochemical, and molecular biology studies clarified the systems and elements necessary for K^+^ transport and showed that their disruption results in hearing loss. This review focuses on recent understandings on the mechanisms underlying the homeostasis of the EP and their pathophysiological relevance.

## Structure and molecular architecture of the lateral cochlear wall

In the lateral wall, the spiral ligament comprises five types of fibrocytes (I∼V), all of which are bathed in the perilymph (Fig. 1a). The stria vascularis is made of three cell types, i.e., marginal, intermediate, and basal cells (Fig. 1b). Epithelial marginal cells and basal cells form monolayers with tight junctions and border the stria from the endolymph and the perilymph, respectively. Intermediate cells, basal cells, and fibrocytes are interconnected by gap junctions and thereby constitute a functional syncytial layer [55, 56, 97]. In this arrangement, it seems probable that the inner parts of these three cell types share the same electrochemical properties and that the basolateral and apical surfaces of the syncytial layer are provided primarily by fibrocytes and intermediate cells, respectively (Fig. 1b) [65, 92, 108]. Taking these considerations together, the lateral wall serves as a double-layered epithelial-like tissue consisting of the marginal cell and syncytial layers (Fig. 1b). Furthermore, endothelial cells of numerous capillaries that penetrate the two layers are also attached to each other by tight junctions [47, 57, 58]. In addition to this observation and to the fact that tight junction networks form in marginal and basal cells, electrically measured input resistance inside of the stria was found to be much higher than the one of the perilymph and the endolymph [74], indicating that this space is morphologically and electrically separated from the two lymphs and the blood. Another important characteristic is the complicated morphology of the intermediate cell membranes and basolateral membranes of marginal cells. These membrane compartments are highly invaginated, so that the extracellular space narrows down to 15 nm [41].

Classical pharmacological experiments using live animals characterized the channels and transporters expressed in the lateral wall and evaluated their possible physiological roles. Vascular and perilymphatic perfusion of ouabain, a blocker of Na^+^,K^+^-ATPases, or loop diuretics such as furosemide and bumetanide, antagonists for the Na^+^,K^+^,2Cl^−^-cotransporter (NKCC), dramatically suppressed the EP, suggesting that these K^+^-uptake transporters act in the stria vascularis and spiral ligament and critically contribute to the EP [38, 39, 62, 63, 66, 69, 90, 109]. Loss of EP was also observed upon systemic injection of Ba^2+^ that inhibits K^+^ channels, but it was not detected by perilymphatic application of this blocker [35, 70]. These results imply that the K^+^ channels expressed in the stria vascularis play key roles in the formation of the EP.

From the 1990s, advanced molecular biology, histochemical, and electrophysiological techniques have unveiled the molecular constituents and cellular and subcellular localizations of channels, transporters, and other apparatuses. It is likely that tight junctions in marginal cell layer contain claudin-1, -2, -3, -8, -9, -10, -12, and -14, and the ones in basal cells consist solely of claudin-11 [58]. Gap junctions included in the syncytial layer are constituted of connexin (Cx)-26, -29, -30, and -43 [55, 56, 67, 96, 114, 118]. Na^+^,K^+^-ATPases in the stria vascularis are complexes of α_1_ and β_2_ subunits and localize on the basolateral membranes of marginal cells, whereas those in the spiral ligament are composed of α_1_ and β_1_ subunits and are expressed on the membranes of fibrocytes (Fig. 1b) [19, 73, 81]. The NKCC in the lateral wall is a type I cotransporter (NKCC1) and is present together with Na^+^,K^+^-ATPases in marginal cells and type II, IV, and V fibrocytes (Fig. 1b) [19, 73]. Furthermore, type I and III fibrocytes bear the K^+^,Cl^−^-transporter KCC3 [11]. We previously demonstrated that an inwardly rectifying K^+^ channel subunit KCNJ10 was expressed in the stria vascularis and responsible for Ba^2+^-sensitive K^+^ channels involved in the generation of EP [35]. Subsequently, another research group found that this channel subunit is localized on the membrane of intermediate cells (Fig. 1b) [4, 98]. In addition, in the marginal cells, the KCNQ1 and KCNE1 subunits were found to assemble and form functional I_KS_ K^+^ channels (KCNQ1/KCNE1) on the apical membrane as well as Cl^−^ channels comprising the α subunit of ClC-Ka or ClC-Kb and the β subunit of barttin (ClC-K/barttin) on the basolateral membrane (Fig. 1b) [27, 77, 82, 88, 101]. The channels and transporters described above are thought to directly or indirectly contribute to unidirectional K^+^ transport across the lateral wall and to the EP formation. Moreover, patch-clamp assays of dissociated strial cells detected nonselective cation (NSC) channels on the basolateral and apical membranes of marginal cells [95, 101, 102]. Apical channels comprise the TRPM4 subunit [83, 113].

On the lateral wall, other ion channels and transporters may be expressed and involved in the control of unidirectional K^+^ transport. Candidates have been obtained by our recent mass spectrometry analysis of membrane proteins extracted from rat stria vascularis [105]. Of 3236 proteins detected in the analysis, 16 ion channels and 62 transporters had not yet been identified in the stria. These include the K^+^ channels KCNJ13 and KCNN4, as well as K^+^,Cl^−^-exchanger family proteins (KCCs). Although it must be carefully evaluated by histochemical approaches whether these K^+^ transport molecules are expressed in the stria vascularis or their detection by mass spectrometry results from the contamination from other tissues, their identification might reveal novel mechanisms of homeostasis of the EP.

## Pathological significance of the molecules expressed in the lateral wall

Intensive research endeavors for the last 20 years have identified that genetic ablations and mutations of ion transport proteins expressed in the stria vascularis result in hearing impairment in mice or humans, as summarized in Table 1. For instance, KCNE1- or KCNQ1-null mice show severe collapse of the scala media and deafness [12, 106], implying that KCNQ1/KCNE1 K^+^ channels are crucial for driving K^+^ transport and maintaining the endolymph. These mice also show vestibular disorder. Loss-of-function mutations of the gene encoding both subunits cause Jarvell and Lange-Nielsen syndrome, accompanied by hearing disorders and arrhythmia. Similar phenotypes were observed in animals lacking the NKCC1-gene [24, 26, 31]. Type 4 Bartter’s syndrome, which is characterized by renal salt loss and hearing impairment, is induced by missense mutations in the amino-terminal region of the Cl^−^ channel subunit barttin [9, 27]. Knockout (KO) of this protein leads to loss of EP in mice [77]. KCNJ10-null mice display an ∼0 mV EP with disruption of the structure of the scala media [71, 79]. Mutations in the gene of this channel subunit underlie the EAST/SeSAME syndrome, characterized by deafness, epilepsy, ataxia, mental retardation, tubulopathy, and electrolyte imbalance [10, 85]. Interest information has been further provided by analyses of the Na^+^,HCO_3_^−^-exchanger SLC26A4, namely, pendrin, and the two-pore domain K^+^ (K_2P_) channel subunit KCNK5. These two proteins are expressed in outer sulcus cells [13, 28, 78], which are also constituents of the lateral wall and localized beside the fibrocytes (Fig. 1a). Mutations in the SLC26A4-gene lead to Pendred syndrome, characterized by abnormal cochlear development, sensorineural hearing loss, and thyroid goiter [18, 49, 87]. Model mice of this disease display phenotypes of cochlear and vestibular dysfunctions with enlarged membranous labyrinths [28, 78]. KCNK5-KO mice exhibit loss of several cell types including outer sulcus epithelial cells and hair cells [13]. In these two mouse lines, the EP was markedly impaired. Therefore, outer sulcus cells may serve as a platform for ion transport involved in the maintenance of the endolymph.

**Table 1:** Genes encoding lateral wall’s ion transport proteins associated with hearing loss in human and mouse

Molecules	Distribution	Human deafness types	Mouse phenotypes (other than deafness)	Reference
KCNE1 (Isk, min K)	MC	Jarvell and Lange-Nielsen syndrome	Collapse of SM and VES, balance disorder	[106]
KCNQ1 (KvLQT1, Kv7.1)	MC	Jarvell and Lange-Nielsen syndrome	Collapse of SM and VES, balance disorder	[12]
KCNJ10 (Kir4.1)	IC	EAST syndrome, SeSAME syndrome	Collapse of SM, loss of EP (∼0 mV)	[10, 71, 79, 85]
KCNK5 (K_2P_5.1)	OSC	–	Loss of EP (∼ +50 mV), degeneration of hair cells and spiral ganglions	[13]
BSND (barttin)	MC	Type 4 Bartter’s syndrome	Loss of EP (∼ +17 mV)	[77]
NKCC1 (SLC12A1)	MC, FC	–	Collapse of SM and VES, balance disorder	[24, 26, 31]
Pendrin (SLC26A4)	SPEC, OSC	Pendred syndrome, DFNB4	Loss of EP (∼0 mV)	[2, 29, 30, 111]
SLC22A4	StE	DFNB60	–	[6]
KCC3 (SLC12A6)	FC	–	Degeneration of hair cells	[11]
GJA1 (Cx43)	FC	AR-nonsydromic deafness	–	[68]
GJB1 (Cx32)	FC	X-linked Charcot-Marie-Tooth and deafness	–	[93]
GJB2 (Cx26)	FC	DFNB1, DFNA3A, hereditary palmoplantar keratoderma with deafness	Loss of EP (∼ +50 mV), degeneration of hair cells	[16, 25, 54, 117, 119]
GJB3 (Cx31)	FC	DFNA2, AR-nonsyndromic deafness	–	[112]
GJB6 (Cx30)	FC	DFNA3B	Loss of EP (∼ +3 mV), degeneration of hair cells	[34, 104]
Panx1	FC, OSC	–	Loss of EP (∼ +60 mV)	[15]

*EP* endocochlear potential, *SM* scala media, *VES* vestibular endolymphatic space, *FCs* fibrocytes, *BCs* basal cells, *ICs* intermediate cells, *MCs* marginal cells, *OSCs* outer sulcus cells, *SPECs* spiral prominence epithelial cells, *StE* strial endothelium, *DFNA* the locus for autosomal dominant nonsyndromic deafness, *DFNB* the locus for autosomal recessive nonsyndromic deafness, *AR* autosomal recessive

KO mice lacking any of the Cx26, 30, and 43 subunits show hearing loss, suggesting the importance of ion transport through the connexin network in the lateral wall [7, 8, 17, 16, 23, 32, 50, 52, 86, 104]. Finally, disruption of the claudin-11-gene results in deafness accompanied by a severe decrease in EP to ∼ +30 mV [33, 57], and it highlights a crucial role for the barrier functions provided by tight junctions of basal cells in the stria vascularis.

## Mechanisms underlying the formation of the EP

Along the identification of the molecular elements that contribute to the EP, the mechanisms underlying this process have been analyzed step by step. Pivotal observations were provided by in vivo electrophysiological experiments performed approximately 30 years ago. Salt et al. inserted a double-barreled K^+^-selective microelectrode, which can simultaneously measure the potential and [K^+^], from the perilymph toward the endolymph across the lateral wall in live guinea pigs [84]. In the stria vascularis, after detecting a cellular compartment exhibiting a high [K^+^], they found a unique extracellular space called “intrastrial space (IS)” that showed a highly positive potential similar to the EP and a low [K^+^] as observed in the perilymph. On the basis of these measurements, they assumed that (1) the cellular compartment close to the IS consisted of basal cells; (2) the IS potential (ISP) was primarily responsible for the EP and formed mainly by K^+^ diffusion potential, i.e., K^+^ equilibrium potential (*E*_*K*_); and (3) this diffusion potential resulted from high K^+^ permeability on the apical surfaces of basal cells. The electrochemical properties of the IS were also confirmed by another report [45]. Later on, patch-clamp and immunohistochemical experiments demonstrated that K^+^ permeability dominated the membranes of intermediate cells, as it emerged from KCNJ10 and other K^+^ channels (Fig. 1b) [4, 100]. According to the aforementioned histological studies [41], the IS corresponds to the 15-nm extracellular space primarily surrounded by invaginated membranes of intermediate and marginal cells (Fig. 1b) [84].

By incorporating these observations, the “two-cell model” and the “five-compartment model” have been proposed to explain the generation of the EP [84, 108]. In this model, the EP represents a potential difference across the lateral wall composed of two layers,

1 $$ EP={v}_{SB}-{v}_{SA}+{v}_{MB}-{v}_{MA,} $$

where *v*_SB_ and *v*_SA_ are the membrane potentials across the basolateral and apical surfaces of the syncytial layer, respectively, and *v*_MB_ and *v*_MA_ are the ones across the basolateral and apical surfaces of the marginal cell layer, respectively (Fig. 1b, c). Each membrane potential was relative to the neighboring extracellular solution, which was defined as 0 mV. Equation 1 indicates that the EP depends on the four membrane potentials provided by the two layers of the lateral wall. Given that K^+^ conductance governs the intermediate cell membranes that correspond to the apical surface of the syncytial layer [100], Eq. 1 can be described by the equation

2 $$ EP\approx {v}_{SB}-\frac{RT}{F} \ln \left(\frac{{\left[{\mathrm{K}}^{+}\right]}_{\mathrm{IS}}}{{\left[{\mathrm{K}}^{+}\right]}_{SY}}\right)+{v}_{MB}-{v}_{MA}. $$

In this scenario, the ISP is equivalent to the transepithelial voltage across the syncytial layer,

3 $$ ISP={v}_{SB}-{v}_{SA}\approx {v}_{SB}-\frac{RT}{F} \ln \left(\frac{{\left[{\mathrm{K}}^{+}\right]}_{\mathrm{IS}}}{{\left[{\mathrm{K}}^{+}\right]}_{SY}}\right). $$

According to patch-clamp analyses and in vivo experiments [72, 84, 99], *v*_SB_, *v*_MB_, and *v*_MA_ were estimated to be small of <10 mV [100]. Therefore, it was assumed that the values of −*v*_SA_, ISP, and EP are approximately equal to each other (Eqs. 2 and 3). This relationship can be described as follows [100]:

4 $$ EP\approx -\frac{RT}{F} \ln \left(\frac{{\left[{\mathrm{K}}^{+}\right]}_{\mathrm{IS}}}{{\left[{\mathrm{K}}^{+}\right]}_{SY}}\right)\approx ISP, $$

where [K^+^]_IS_ and [K^+^]_SY_ are the [K^+^] in the IS and inside the syncytial layer, respectively. We assayed the lateral wall of live guinea pigs with double-barreled K^+^-selective microelectrodes and confirmed the aforementioned potential and [K^+^] in the IS, as shown in Fig. 1c [1, 74, 116]. Our measurements also determined the properties of other compartments. First, the potential inside the syncytial layer, which is equivalent to *v*_SB_ in Eqs. 1–3, is moderately positive (∼ +7 mV), compared to the perilymph. Second, there is only a slight transepithelial voltage across the marginal cell layer (i.e., *v*_MB_ − *v*_MA_; see Eqs. 1 and 2), and thereby, the ISP is similar to the EP. These observations reinforce the idea that the ISP represents the K^+^ diffusion potential and dominates the EP, as described by Eq. 5.

**Fig. 2:**
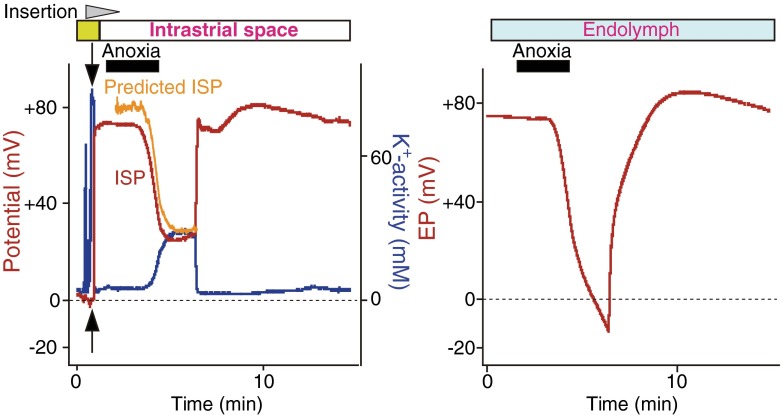
Effects of anoxia on the electrochemical properties of IS and EP. In the *left panel*, a double-barreled microelectrode sensitive to potential (*red*) and K^+^ (*blue*) was inserted in vivo from the perilymph (outset of the traces) toward the endolymph across the lateral wall of cochlea during the time interval marked with the *wedge filled with gray* (*top of the panel*). K^+^ concentration is indicated as K^+^ activity (*a*K^+^). Location of the tips of the electrodes is shown by *bars at the top of the panels*. After passing the syncytial layer (*green bar* above the trace; see also Fig. 1c), the electrode encountered the intrastrial space (IS). The EP was simultaneously monitored with a different glass microelectrode placed in the endolymph (*right panel*). Anoxia was imposed on the animal during the period indicated by *bars filled with black* above the traces. The values of the IS potential (ISP) were calculated from the measurements of *a*K^+^ in the IS (*a*K^+^_IS_) and *a*K^+^ and the potential of the syncytial layer (*a*K^+^_SY_ and *v*_SB_, *arrows in the left panel*) with the equation ISP = *v*_SB_ + RT/F ln(*a*K^+^_IS_/*a*K^+^_SY_). The predicted ISP (*orange*) was in agreement with the measured ISP (*left panel*). Note that the EP (*right panel*) was suppressed more strongly than the ISP (*left panel*) during anoxia. Reproduced and modified from [74]

**Fig. 3:**
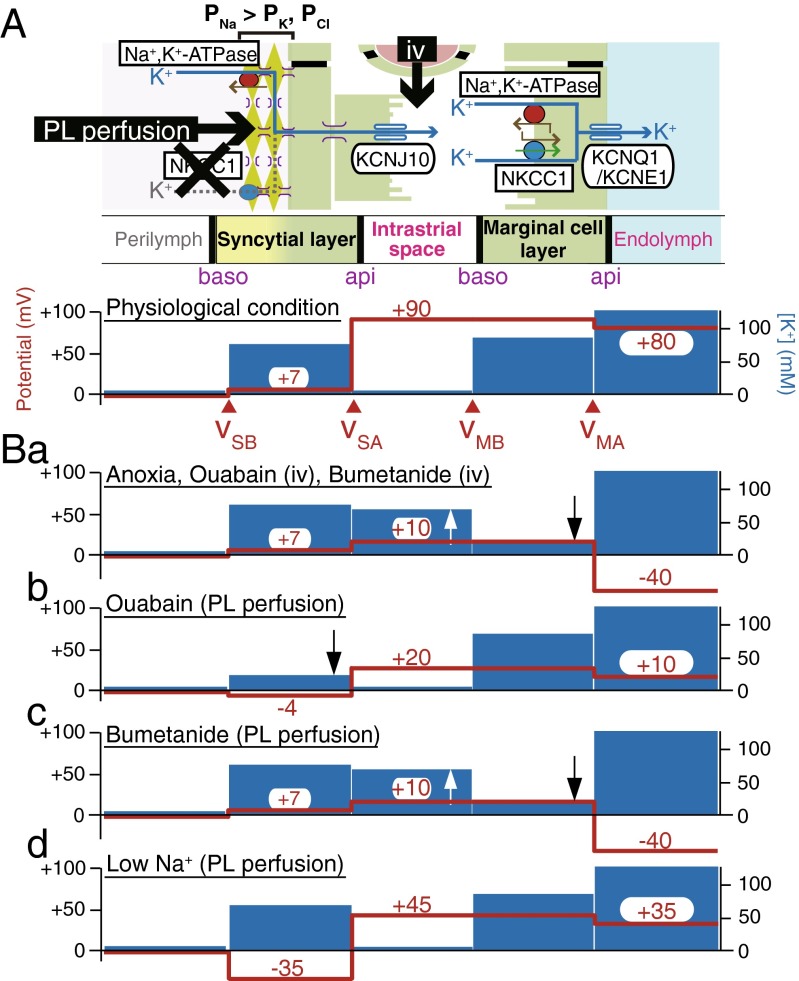
Summary profiles of the electrochemical properties of the lateral wall. In **A**, the *upper panel* illustrates the cellular compartments of the lateral wall and the channels and transporters contributing to unidirectional K^+^ transport and EP; the *lower panel* depicts the potential (*red*) and [K^+^] (*blue*) in intracellular and extracellular spaces under physiological conditions. Intravenous application (iv) of artificial solutions containing chemical compounds or modified ion compositions may primarily affect channels and transporters on the apical surface (*api*) of the syncytial layer and on the basolateral surface (*baso*) of the marginal cell layer, whereas perilymphatic (PL) perfusion is likely to strongly influence proteins on the basolateral surface of the syncytial layer. *v*_SB_, *v*_SA_, *v*_MB_, and *v*_MA_ indicate the membrane potentials across the basolateral and apical surfaces of the syncytial layer and across the basolateral and apical surfaces of the marginal cell layer, respectively. The panels in **B** display the potential and [K^+^] properties observed during anoxia, vascular perfusion of ouabain or bumetanide (*a*), perilymphatic perfusion of ouabain (*b*), bumetanide (*c*), and a low Na^+^ solution (*d*). Observation of *b*, *c*, and *d* implies that the Na^+^,K^+^,2Cl^−^-cotransporter type 1 (NKCC1) present on the basolateral surface of the syncytial layer is unlikely to transport K^+^ and that this membrane domain is more permeable to Na^+^ than K^+^ and Cl^−^ (*P*_Na_ *> P*_K_, *P*_Cl_), as shown in the *upper panel* of **A** [115, 116]. The *upward* and *downward arrows* indicate the increase and decrease of [K^+^]. Reproduced and modified from [1, 74, 115, 116]

Under physiological conditions, [K^+^]_SY_ greatly exceeds [K^+^]_IS_ (Fig. 1c), providing a highly positive potential (∼ +80 mV) for the ISP and EP (Eq. 4). On the other hand, when Na^+^,K^+^-ATPases or NKCC in the stria vascularis were impaired by anoxia, which would mimic ischemia, or by vascular perfusion of blockers, the EP dramatically reduced to −30 to −40 mV [59]. Under these conditions, it is plausible that [K^+^]_SY_ is smaller than [K^+^]_IS_ (Eq. 4) or that *v*_SB_, *v*_MB_, or *v*_MA_ is significantly modulated (Eq. 2). However, these alternations were not considered in the two-cell model or five-compartment model. These issues were addressed by our fine in vivo measurements [74]. In the example shown in Fig. 2, a K^+^-selective microelectrode was held in the IS, while the other electrode was being inserted in the endolymph to continuously monitor the EP. Imposition of anoxia on the animal caused an increase in [K^+^]_IS_ and a decrease in ISP. Importantly, the ISP calculated from Eq. 3 with the measured [K^+^]_IS_, [K^+^]_SY_, and *v*_SB_ (constant) was in agreement with the ISP directly recorded with the K^+^-selective microelectrode. This finding supports that the ISP stems primarily from the K^+^ diffusion potential. On the other hand, the simultaneously measured EP was reduced more strongly than the ISP and reached a negative value. This indicates that anoxia can enlarge the transepithelial voltage across the marginal cell layer (*v*_MB_ − *v*_MA_) and that this property is involved in the reduction of the EP. To clarify the underlying mechanisms, we examined marginal cells and found that a large K^+^ diffusion potential was induced on the apical surface of the marginal cell layer and it formed the difference between the EP and ISP (Figs. 3A, Ba). Under physiological conditions, the marginal cell K^+^ diffusion potential, which is likely to emerge from KCNQ1/KCNE1 channels (Fig. 1b), is relatively small, because of the similarity between [K^+^] in the marginal cell layer ([K^+^]_MC_) and [K^+^] in the endolymph ([K^+^]_EL_) (Fig. 3A). Application of anoxia markedly decreased [K^+^]_MC_ with a minimal effect on [K^+^]_EL_, increasing the diffusion potential (Fig. 3Ba) [74]. Little change was detected in the membrane potential across the basolateral surface of the marginal cell layer (*v*_MB_), in spite of the elevation of [K^+^]_IS_ and the reduction of [K^+^]_MC_. This experimental observation is reasonable, given that patch-clamp analysis of isolated marginal cells detected abundant Cl^−^ conductance but little K^+^ conductance on their basolateral membranes [88]. Similar results were obtained when ouabain or the loop diuretic bumetanide was vascularly applied (Fig. 3Ba) [74]. In summary, the EP primarily depends on two K^+^ diffusion potentials on the apical surfaces of the syncytial and marginal cell layers,

5 $$ \begin{array}{c}\hfill EP\approx {v}_{SB}-\frac{RT}{F} \ln \left(\frac{{\left[{\mathrm{K}}^{+}\right]}_{\mathrm{IS}}}{{\left[{\mathrm{K}}^{+}\right]}_{SY}}\right)+{v}_{MB}-\frac{RT}{F} \ln \left(\frac{{\left[{\mathrm{K}}^{+}\right]}_{\mathrm{EL}}}{{\left[{\mathrm{K}}^{+}\right]}_{MC}}\right)\hfill \\ {}\hfill \approx {v}_{\mathrm{SB}}-\frac{RT}{F} \ln \left(\frac{{\left[{\mathrm{K}}^{+}\right]}_{\mathrm{IS}}}{{\left[{\mathrm{K}}^{+}\right]}_{\mathrm{SY}}}\right)-\frac{RT}{F} \ln \left(\frac{{\left[{\mathrm{K}}^{+}\right]}_{\mathrm{EL}}}{{\left[{\mathrm{K}}^{+}\right]}_{\mathrm{MC}}}\right).\hfill \end{array} $$

## Integration of circulation current into processes for formation of the EP by theoretical approaches

Under physiological conditions, [K^+^]_IS_ is lower than [K^+^]_MC_ (Figs. 1c and 3A). However, blocking Na^+^,K^+^-ATPases or NKCC1 in marginal cells, [K^+^]_IS_ and [K^+^]_MC_ gradually change, and finally, the former exceeds the latter (Fig. 3Ba) [37, 74]. These [K^+^] dynamics, which underlie the reduction of ISP and EP, may derive from the modulation of currents through channels and transporters in the lateral wall. Nevertheless, such currents are neither measurable directly by in vivo electrophysiological approaches nor described in previous models and equations. Therefore, clarification of this issue requires theoretical approaches.

Each of the marginal cell and syncytial layers expresses particular K^+^ channels or K^+^-uptake transporters on different surfaces (Fig. 1b). Assembly of these two layers in vivo must allow K^+^ to unidirectionally move from the perilymph into the endolymph across the lateral wall. MET channels in hair cells are slightly open even in their resting state [80], so that in vivo K^+^ constantly flows through cells. Taking these considerations together, it is plausible that, even without acoustic stimulation, K^+^ is transported from the perilymph to the endolymph and then returns to the endolymph through the three layers in the cochlea, i.e., the syncytial, marginal cell, and hair cell layers (Fig. 1a). This “circulation current” composed of K^+^ [75], also referred to as “K^+^ recycling” [55, 56, 109, 110], likely corresponds to an idling current in the electrical circuit. The existence of a circulation current was first proposed in Davis’ battery theory [21], and it was validated by in vivo recording of the voltage gradient representing ion flows with a microelectrode in the perilymph [120]. The circulation current has been suggested to be involved in the maintenance of high potential and [K^+^] in the endolymph [20, 55, 56, 109, 110].

**Fig. 4:**
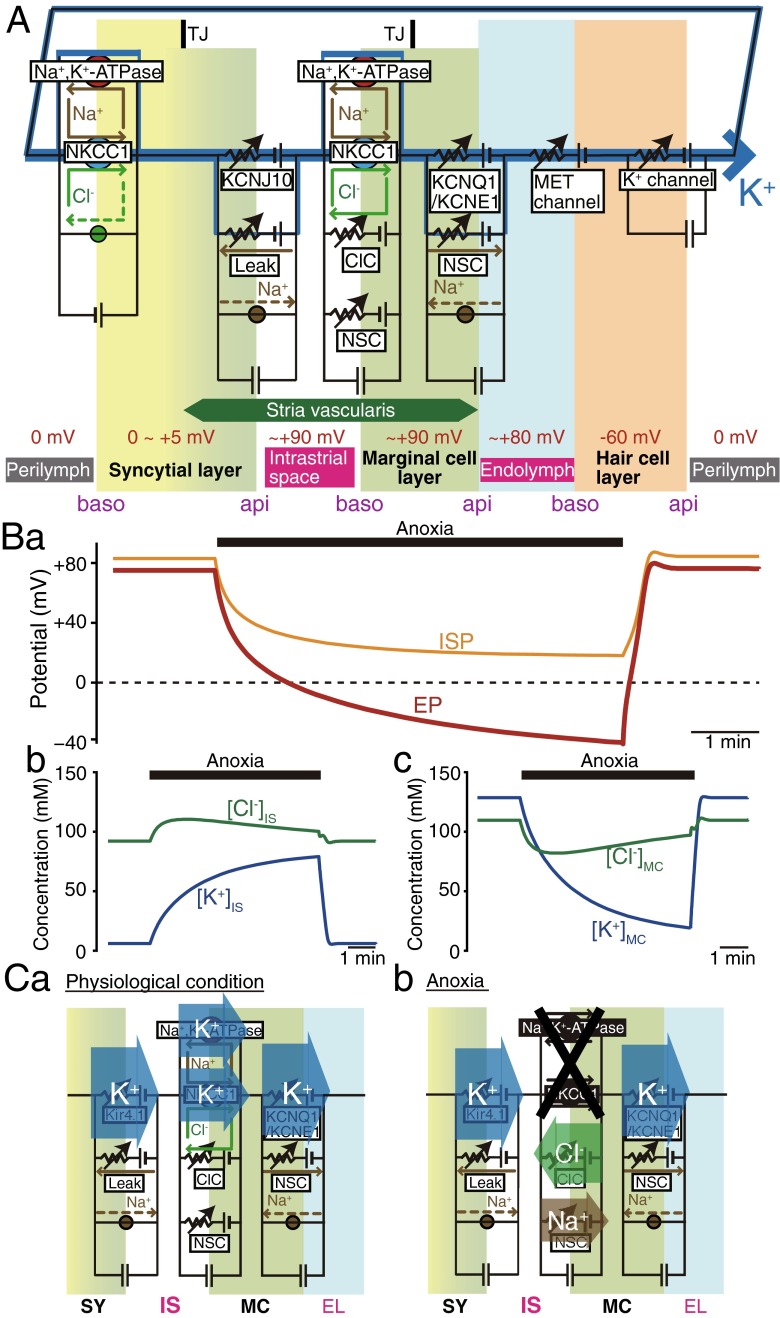
Computer simulation of electrochemical dynamics in the cochlea. **A** Key elements in the Nin-Hibino-Kurachi (NHK) model. The profile of each membrane is described by electrical circuits that represent the function of channels and transporters. The circuits in six membrane domains deriving from the syncytial, marginal cell, and hair cell layers were connected in series to allow the flow of circulation current, which is carried by K^+^ under physiological conditions. *NKCC1* Na^+^,K^+^,2Cl^−^-cotransporter type 1, *ClC* ClC-K/barttin type Cl^−^ channel, *NSC* nonselective cation channel, *TJ* tight junction, *MET* mechanoelectrical transduction, *baso* basolateral, *api* apical. **B** Reproduction of electrochemical properties in the cochlea under physiological and anoxic conditions. Change of endocochlear potential (EP; *a*), IS potential (ISP; *a*), and ion concentrations in the IS (*b*; [K^+^]_IS_ and [Cl^−^]_IS_) and marginal cell layer (*c*; [K^+^]_MC_ and [Cl^−^]_MC_) were obtained by using the NHK model as reported in [75]; these results are in good agreement with in vivo measurements. Note that [Cl^−^] is moderately altered during anoxia (*b* and *c*). To simulate anoxic conditions, the activities of Na^+^,K^+^-ATPases and NKCC1 in the basolateral surface of the marginal cell layer were reduced to 5 % of the control. **C** Profiles of the circulation current in the lateral wall. Net flow of each ion across the apical surface of the syncytial layer (SY), as well as through the basolateral and apical surfaces of the marginal cell layer (MC), are calculated and displayed in the *panels*. Under physiological conditions, the amplitude of Na^+^ outflux through Na^+^,K^+^-ATPases is set to match the one of Na^+^ influx through NKCC1. Cl^−^ transport by NKCC1 also cancels Cl^−^ flow across the ClC-K/barttin-type Cl^−^ channel (ClC). Due to these arrangements, the circulation current is composed purely of K^+^ (*a*). Under anoxic conditions (*b*), the carrier of the circulation current in the basolateral surface of the marginal cell layer switches from K^+^ to Cl^−^ and Na^+^ that permeate the ClC and the nonselective cation channel (NSC), respectively. This change causes an increase in [K^+^]_IS_ and [Cl^−^]_IS_ and a decrease in [K^+^]_MC_ and [Cl^−^]_MC_, as shown in B *b* and B *c*. *IS* intrastrial space, *EL* endolymph. Reproduced and modified from [75]

The conditions of cells controlled by circulation current are different from the ones of cells isolated from the organism. When isolated cells are bathed in artificial extracellular solution filling an experimental chamber, their resting membrane potential (RMP) can be predicted with the Nernst or Goldman-Hodgkin-Katz (GHK) equations. In this “equilibrium state,” the total current across the cell membrane is zero. This is the principle of the two-cell model or five-compartment model (Eqs. 1–4) [20, 84, 100] as well as the one of our earlier model (Eq. 5) [37, 74]. On the other hand, in vivo, the unidirectional circulation current is likely to constantly flow across the three layers even under physiological conditions. In this case, membrane potentials as well as ion concentrations in every cell are in the “steady state” and therefore constant. Based on this idea, we developed a computational model, the “Nin-Hibino-Kurachi (NHK) model,” to quantitatively describe the dynamics of the electrochemical properties of the cochlea (Fig. 4A) (for detailed equations, see [75]). In this model, the profile of each membrane is characterized by equivalent circuits of Hodgkin-Huxley-type equations that represent the function of channels and transporters. The circuits in six membrane domains were then connected in series to reconstitute the circulation current. In other words, the circulation current corresponds to the sum of the currents through all the channels and transporters in each membrane domain. This arrangement allows the circulation current to control membrane potentials as well as ion concentrations in the extracellular and intracellular compartments. In particular, the latter parameter is described by the following equation:

6 $$ \frac{d\left[X\right]}{dt}=\frac{I_{X,\mathrm{in}}-{I}_{X,\mathrm{out}}}{V\times F}, $$

where [*X*] is the concentration of ion X, *I*_*X*,in_ is the inward current of X, *I*_*X*,out_ is the outward current of X, *V* is the volume of the extracellular or intracellular compartment, and *F* is the Faraday constant. Each of *I*_*X*,in_ and *I*_*X*,out_ is the sum of the current passing through an X-permeable channel and the X-flow through the transporter carrying X on the membrane. Note that *I*_*X*,in_ and *I*_*X*,out_ are components of the circulation current. Finally, the current passing through MET channels always equals the circulation current in the route across the hair cell layer. Thus, the amplitude of the circulation current depends on the potential difference across the apical surface of hair cells, i.e., the difference between the EP and the potential inside hair cells with respect to the perilymph. To summarize, under physiological conditions, the amplitude of the circulation current is constant and flows through every membrane of the three layers; *I*_*X*,in_ is same as *I*_*X*,out_ for every compartment (Eq. 6). This situation provides constant values for all of the ionic concentrations (*d*[*X*]/*dt* = 0), all of the membrane potentials, and the EP. However, when functions of channels or transporters are perturbed, the circulation current changes, modulating ionic concentrations and membrane potentials.

By using the NHK model, we simulated the electrochemical profile in the lateral wall and endolymph under physiological conditions and during anoxia that mimics ischemic conditions. Our in vivo experiments strongly suggest that imposition of anoxia on animals inhibited not only Na^+^,K^+^-ATPases but also NKCC on the basolateral surface of the marginal cell layer [75]. The measured behaviors for EP, ISP, [K^+^]_IS_, and [K^+^]_MC_ were highly reproducible in silico, when the activities of both of these K^+^-uptake transporters were reduced to 5 % of the control (Fig. 4B). Simulated changes of current properties in the lateral wall are schematically illustrated in Fig. 4C [75]. Under physiological conditions, the circulation current is set to be purely made up of K^+^ at any membrane domain (Fig. 4Ca). During anoxia, marginal cell transporters, which are blocked as mentioned above, can barely transport K^+^. Instead, the circulation current can pass as flows of Cl^−^ and Na^+^ through the ClC-K/barttin and NSC channels, respectively; both of which are coexpressed with the two K^+^-uptake transporters. This indicates that the carrier of the circulation current can switch from K^+^ to other ions on the basolateral surface of the marginal cell layer (Fig. 4Cb). Consequently, K^+^ influx exceeds K^+^ efflux in the IS and vice versa in the marginal cell layer, causing an increase in [K^+^]_IS_ and a decrease in [K^+^]_MC_ (Fig. 4Bb and Bc). In the late phase of anoxia, [K^+^]_IS_ significantly exceeds [K^+^]_MC_ (Fig. 3Ba). These changes gradually affect membrane potentials and therefore reduce the ISP and EP (Fig. 4Ba). This EP impairment further results in the reduction of the amplitude of the circulation current.

## Profile of the basolateral surface of the syncytial layer

The EP is determined by the potentials across the four membrane domains of the lateral wall, i.e., *v*_SB_, *v*_SA_, *v*_MB_, and *v*_MA_ (see Eqs. 1 and 5 and Fig. 3A, Ba). As described above, the marginal cell apical and basolateral surfaces as well as the syncytial layer apical surface, which corresponds to the intermediate cell membrane, have been so far considerably characterized by multiple approaches. However, little is known about the functional elements of the syncytial layer basolateral surface, formed primarily by fibrocytes of the spiral ligament [108].

Earlier studies demonstrated that perfusion of either ouabain or loop diuretics to the perilymph, in which fibrocytes are immersed, dramatically reduces the EP [69, 90]. Therefore, among several proteins expressed in fibrocytes, Na^+^,K^+^-ATPases and NKCC seem to regulate electrochemical properties and K^+^ transport in the lateral wall (Fig. 1b). This idea was tested in our in vivo experiments with K^+^-selective double-barreled microelectrodes. Perilymphatic application of ouabain markedly decreased [K^+^]_SY_ with a minimum change of [K^+^]_IS_ [1] (Fig. 3Bb). As a consequence, the ISP and the EP declined (Eqs. 3 and 5). On the other hand, whereas bumetanide was perfused to the perilymph, [K^+^]_SY_ barely changed [116] (Fig. 3Bc). These results strongly suggest that, in the syncytial layer, Na^+^,K^+^-ATPases, but not NKCC1, functionally transport K^+^ across the basolateral surface and maintain a high [K^+^]_SY_, essential for the large K^+^ diffusion potential on the apical surface. Further analyses revealed that during perilymphatic perfusion of bumetanide, [K^+^]_IS_ increases and [K^+^]_MC_ decreases, as observed during vascular perfusion [116] (Fig. 3Bc). Therefore, this compound is likely to reach the NKCC1 on the basolateral surface of the marginal cell layer and impair the ISP and EP in processes similar to anoxia (see Eq. 5 and Fig. 3Ba).

It is surprising that in vivo and under physiological conditions, the basolateral surface of the syncytial layer exhibits a significantly positive potential (∼ +7 mV) with respect to the perilymph (*v*_SB_ = ∼ +7 mV; Figs. 1c and 3A) [116], although in general, eukaryotic cells show a negative membrane potential at their resting state [40, 48, 76]. The positive RMP of the syncytial layer is critical for harvesting the K^+^ diffusion potential on the apical surface to set the ISP and EP to a highly positive value (Eqs. 3 and 5), which is essential for hearing. As already mentioned, the syncytial basolateral surface is mainly provided by fibrocytes in the spiral ligament. In spite of the physiological importance of fibrocytes, the machineries underlying the establishment of this unique RMP have not yet been characterized. A major reason is likely to be the technical difficulty of patch-clamp assays conducted on fibrocytes tightly embedded in the extracellular matrix [53]. Our in vivo experiments with double-barreled K^+^-selective microelectrodes demonstrated that *v*_SB_ is remarkably hyperpolarized by perilymphatic perfusion of a solution with low [Na^+^] (Fig. 3Bd), but it can only moderately be changed by a solution containing either high [K^+^] or low [Cl^−^] [115]. These observations imply that the fibrocyte membrane is more permeable to Na^+^ than to K^+^ and Cl^−^ (Fig. 3A), and this unusual profile is critically involved in establishment of the positive RMP under physiological conditions. During perfusion of the low-Na^+^ solution, the ISP and EP were reduced to an extent similar to the alternation of *v*_SB_ (Fig. 3Bd), suggesting that this perturbation minimally affects the other membrane potentials in the lateral wall.

## Conclusions and perspectives

As described above, various experimental techniques have demonstrated the molecular and physiological architectures that drive unidirectional K^+^ transport across the lateral wall. This ionic flow is likely to be involved in maintaining the electrochemical properties of the syncytial and marginal cell layers and control the two K^+^ diffusion potentials that form the EP. Correlation of the circulation current, which underlies K^+^ transport across the lateral wall, with the EP has also been demonstrated by theoretical approaches. In spite of these achievements, several issues remain to be clarified. First, it will be necessary to identify the proteins responsible for Na^+^ permeability, which plays a key role in maintaining the positive RMP in fibrocytes. A second interesting objective would be to identify the triggers that activate the fibrocyte NKCC1, a transporter that is likely to be silent under physiological conditions. The third open question is the process by which KCC3 in fibrocytes as well as SLC26A4 and KCNK5 in outer sulcus cells regulate the EP. Fourth, some of the channels and transporters in the lateral wall are expected to be functionally affected by Ca^2+^ or H^+^, but the relevance of these ions to the EP is still elusive. This issue may require detailed information on several Ca^2+^ or H^+^ transporting molecules detected by our mass spectrometry experiments conducted on strial proteins [105]. All of these analyses will enable us to improve the NHK model and thereby more precisely describe how the EP and circulation current interplay with each other. A combination of improved computational models and conventional experimental approaches will help us to theoretically understand the reactions of the EP and the properties of the lateral wall in response to different acoustic stimuli, as well as to identify the pathophysiological mechanisms underlying hearing impairment observed in various human diseases and animal models. Further research will contribute to advance the auditory science and medical therapies for hearing disorders.
